# Physical Demands of Top-Class Soccer Friendly Matches in Relation to a Playing Position Using Global Positioning System Technology

**DOI:** 10.1515/hukin-2015-0073

**Published:** 2015-10-14

**Authors:** Javier Mallo, Esteban Mena, Fabio Nevado, Víctor Paredes

**Affiliations:** 1 Sports Department, Physical Activity and Sport Sciences Faculty, Technical University of Madrid.; 2 University of Castilla la Mancha.; 3 Autónoma University of Madrid.; 4 Camilo José Cela University of Madrid. Rayo Vallecano de Madrid.

**Keywords:** Association football, GPS, high-intensity activities, accelerations, heart rate

## Abstract

The aim of this study was to examine the physical demands imposed on professional soccer players during 11-a-side friendly matches in relation to their playing position, using global positioning system (GPS) technology. One hundred and eleven match performances of a Spanish “La Liga” team during the 2010–11 and 2011–12 pre-seasons were selected for analysis. The activities of the players were monitored using GPS technology with a sampling frequency of 1 Hz. Total distance covered, distance in different speed categories, accelerations, and heart rate responses were analyzed in relation to five different playing positions: central defenders (n=23), full-backs (n=20), central midfielders (n=22), wide midfielders (n=26), and forwards (n=20). Distance covered during a match averaged 10.8 km, with wide and central midfielders covering the greatest total distance. Specifically, wide midfielders covered the greatest distances by very high-intensity running (>19.8 km·h-1) and central midfielders by jogging and running (7.2–19.7 km·h-1). On the other hand, central defenders covered the least total distance and at high intensity, although carried out more (p<0.05–0.01) accelerations than forwards, wide midfielders, and fullbacks. The work rate profile of the players obtained with the GPS was very similar to that obtained with semi-automatic image technologies. However, when comparing results from this study with data available in the literature, important differences were detected in the amount of distance covered by sprinting, which suggests that caution should be taken when comparing data obtained with the GPS with other motion analysis systems, especially regarding high-intensity activities.

## Introduction

Traditional time-motion analysis systems have been replaced during the last years by semi-automatic computerized player tracking technologies which are currently used at the elite European club level (Carling et al., 2008). The use of these image recognition systems allows tracking the movements of all the players during a match, helping to build databases which include relevant physical performance indicators from the players of a squad. This match day assessment has been complemented with the measurement of physical parameters during training by means of global positioning system (GPS) technology, which represents a portable and economic procedure of monitoring workloads (Witte and Wilson, 2004; MacLeod et al., 2009). In addition, the GPS is able to provide almost immediate feedback to coaches straight after the training session concludes. Altogether, the strategy of evaluating physical performance in competition with semi-automatic video capture systems and physical training workloads with the GPS is a current common procedure employed by sports scientists and performance analysts in elite soccer. However, Randers et al. (2010) compared four different match analysis systems concluding that physical data obtained with different methodologies should not be interchangeable. Complementarily, Harley et al. (2011) reported similar findings when comparing data obtained by a semi-automatic video capture system and GPS devices, as significant differences were detected in total distance covered and by high-intensity running and sprinting. This might imply that results can be influenced by the motion analysis system employed in the data recording.

The physical demands imposed on top-class soccer players have been extensively documented during the last years (Bradley et al., 2009; Dellal et al., 2011; Di Salvo et al., 2007; Rampinini et al., 2007). From all of these studies it has been concluded that, on average, a player covers around 11 km during a game. However, due to intermittent nature of the play, total distance covered per se represents an insufficient parameter to understand the overall physical requirements and, thus, distance covered at high speeds seems to be a better performance indicator and has been related to the standard of the competition (Di Salvo et al., 2009; Mohr et al., 2003; Randers et al., 2007). Additionally, physical demands are affected by the positional role of the soccer players in the playing formation of the team. Consequently, central midfielders run the greatest distances during the games, whereas wide midfielders cover the greatest distances at high intensities (Di Salvo et al., 2007, 2009; Mohr et al., 2003). To our knowledge, physical requirements determined with the GPS on 11-aside soccer games involving professional players have not been documented so far. It would be expected that physical parameters would follow a similar pattern as those registered with video-analysis methodologies, but with different absolute values. Hence, if training loads are monitored by means of the GPS, it would be interesting to have match day position specific data obtained with the GPS to which to compare with.

The amount of high-intensity exercise performed by the players has been identified as the activity exceeding a certain speed threshold (Di Salvo et al., 2009; Mohr et al., 2003; Randers et al., 2007). However, this kind of exercise should also include phases where the players are not running intensively, but are exposed to sudden changes of speed (accelerations) despite moving at initial lower velocities as it frequently happens during match play. Current semi-automatic video capture systems do not provide acceleration data and, therefore, information about accelerations in soccer is limited and, to our knowledge, has only been reported from Australian matches (Varley et al., 2011). It appears important to examine if elite European soccer players perform a similar number of accelerations during a match as Australian soccer players do.

Thus, the aim of this study was to examine the physical demands imposed on professional soccer players during 11-a-side friendly matches in relation to their playing position using GPS technology. An additional objective was to determine the number of accelerations carried out by the players in the course of the matches.

## Material and Methods

### Participants

One hundred and eleven match performances of a Spanish “La Liga” (national highest competitive level) team were selected for analysis. All the measurements were carried out during 17 pre-season 90-min friendly matches prior to the commencement of the 2011–12 (*n*=9) and 2012–13 (*n*=8) season. The soccer players (mean ± *SD*, age: 24.8 ± 4.2 years; body height: 180.1 ± 5.2 cm; body mass: 75.2 ± 5.1 kg) were classified according to their positional role in the playing formation of the team (Bradley et al., 2009; Di Salvo et al., 2007) into central defenders (CD; *n*=23), full-backs (FB; *n*=20), central midfielders (CM; *n*=22), wide midfielders (WM; *n*=26), and forwards (FW; *n*=20). All the players were fully informed of all experimental procedures before giving their written informed consent to participate. The study was approved by the Ethics Committee of the University of Madrid and conformed to the Declaration of Helsinki.

### Procedures

This study employed a descriptive design to examine the activity profiles of professional soccer players. As the use of the GPS in soccer competitive games is currently restricted by the Laws of the Game, physical demands were determined on the basis of friendly matches. The GPS has been extensively used during the last years in a team sports environment, as they provide a valid and reliable way of estimating physical training workloads (Barbero-Álvarez et al., 2010; Coutts et al., 2010; McLellan et al., 2011; Randers et al., 201). Additionally, the GPS enables studying physical variables which have an impact on the amount of high-intensity exercise performed by soccer players, as the frequency and intensity of accelerations.

The activities of the players were monitored using GPS technology (SPI Elite, GPSports Systems, Camberra, Australia) with a sampling frequency of 1 Hz. Before the beginning of the warm-up, the GPS units were switched on and placed on the upper back of the players, using a neoprene harness to avoid its movement during the exercise. The GPS unit integrated a triaxial *(x, y, z*) accelerometer which captured data at 100 Hz and allowed determining the changes of speed in the displacements of the players. In addition, the heart rate was monitored synchronously by means of short range telemetry in 1 s intervals during the matches (Polar Electro Oy, Kempele, Finland). At the end of the matches, all data were downloaded to a laptop and treated using the software Team AMS V2.1 (GPSports, Camberra, Australia).

The activities carried out by the players were classified into categories according to the following speed thresholds (after Bradley et al., 2009): (i) standing still (0–0.6 km·h^−1^), (ii) walking (0.7–7.1 km·h^−1^), (iii) jogging (7.2–14.3 km·h^−1^), (iv) running (14.4–19.7 km·h^−1^), (v) high-speed running (19.8–25.1 km·h^−1^), and (vi) sprinting (>25.1 km·h^−1^). High-intensity running consisted of running, high-speed running, and sprinting (running speed >14.4 km·h^−1^), whereas very high-intensity running represented the sum of high-speed running and sprinting (running speed >19.8 km·h^−1^). Maximal running speed was defined as the peak speed a player reached during the match. The frequency of accelerations was computed into the following categories (after Cunniffe et al., 2009): (i) <1.0 m·s^−2^, (ii) 1.1–1.5 m·s^−2^, (iii) 1.6–2.0 m·s^−2^, (iv) 1.6–2.0 m·s^−2^, and (v) >2.5 m·s^−2^. After Aughey (2010) the number of maximal accelerations (>2.78 m·s^−2^) were also included in the analysis. Finally, the heart rate was expressed in relation to the individual maximal heart rate (%HR_max_) of the participants and classified into the following categories (after Cummins et al., 2013): (i) <60% HR_max_, (ii) 61–70% HR_max_, (iii) 71–80% HR_max_, (iv) 81–90% HR_max_, (v) 91–95% HR_max_, and (vi) >95% HR_max_. As players used heart rate monitors during matches, fitness tests, and training sessions, peak values reached in any of these conditions were determined as the individual HR_max_.

The validity and reliability of the GPS for match analysis have been reported in previous studies (Randers et al., 2010). Specifically, McLellan et al. (2011) assessed the reliability of the actual GPS model reporting <3% and <5.5% variations in total distance and speed, respectively. In addition, Coutts and Duffield (2010) determined the coefficient of variation (CV) for total distance at 3.6% and for peak speed at 2.3%, whereas the CV for repeated sprints was established at 1.7% (Barbero-Álvarez et al., 2010).

### Statistical Analysis

All statistical analyses were conducted using SPSS for Windows version 18.0 (SPSS Inc., Chicago, IL, USA). The normality distribution of the data was checked using the Kolmogorov-Smirnov test and homogeneity of variance was assessed by the Hartley test. Differences between playing positions were tested with one-way analysis of variance (ANOVA). When a significant difference occurred, Tukey’s *post-hoc* tests were used. The significance of the difference between two means was determined by the effect size, with values of 0.2, 0.5, and 0.8 representing small, medium, and large differences, respectively (Cohen, 1998). Statistical significance was set at *p* < 0.05. Data are reported as means and *SD*.

## Results

### Distances covered

Total distance covered during a match averaged 10793 ± 1153 m, with WM covering more (*p* < 0.05) distance than CD (11321 ± 1238 m vs. 10206 ± 1067, respectively; effect size: 0.96). Table 1 shows distance covered at different speeds in relation to the playing position. Distance covered while jogging by CM was greater (*p* < 0.05) than by FB (effect size: 1.26) and FW (effect size: 1.03). In addition, CM covered greater (*p* < 0.01) distances by running than FB (effect size: 1.57) and CD (effect size: 1.38).

**Figure 1:**
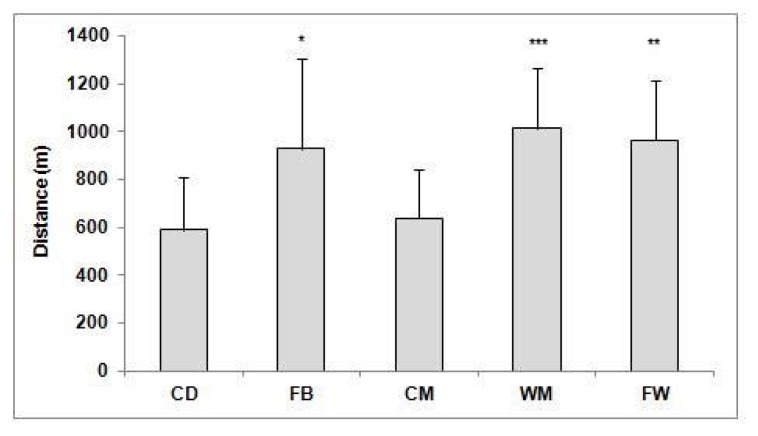
Distance covered (m) by very high-intensity running during a match in relation to a playing position ^*^Significant difference (p < 0.05) between FB and CD and CM. ^**^Significant difference (p < 0.01) between FW and CD and CM. ^***^Significant difference (p < 0.001) between WM and CD and CM. CD: Central Defenders. FB: Fullbacks. CM: Central Midfielders. WM: Wide Midfielders. FW: Forwards.

Distance covered running at speeds exceeding 14.4 km·h^−1^ averaged 2548 ± 636 m. Central defenders covered less distance by high-intensity running (2074 ± 561) than FW (2681 ± 419; *p* < 0.05; effect size: 1.22), central (2697 ± 565 m; *p* < 0.05; effect size: 1.11), and wide (2881 ± 667 m; *p* < 0.001; effect size: 1.30) midfielders. Full-backs covered 2364 ± 599 m by high-intensity running. Figure 1 illustrates distance covered by very high-intensity running, which averaged 822 ± 320 m. Central defenders (591 ± 218 m) and CM (640 ± 206 m) covered less distance by very high-intensity running than WM (1016 ± 252 m; *p* < 0.001; effect size: 1.80 and 1.62, respectively), FW (966 ± 247 m; *p* < 0.01; effect size: 1.62 and 1.44, respectively), and FB (931 ± 375 m; *p* < 0.05; effect size: 1.13 and 0.98, respectively).

**Figure 2:**
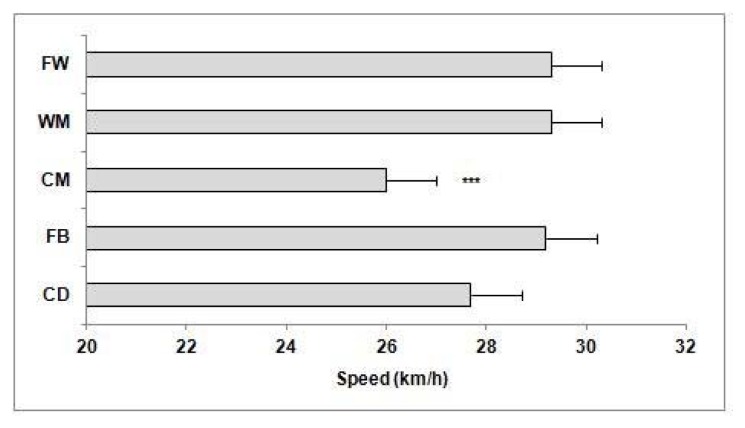
Maximal running speed (km·h^−1^) during a match in relation to a playing position Note: ^***^Significant difference (p < 0.001) between CM and FB, WM and FW. CD: Central Defenders. FB: Fullbacks. CM: Central Midfielders. WM: Wide Midfielders. FW: Forwards.

Maximal running speed during the match averaged 28.3 ± 2.5 km·h^−1^. Central midfielders achieved lower (*p* < 0.001) peak speeds (26.0 ± 2.1 km·h^−1^) than FB (29.2 ± 2.7 km·h^−1^; effect size: 1.34), WM (29.3 ± 2.3 km·h^−1^; effect size: 1.49), and FW (29.3 ± 2.0 km·h^−1^; effect size: 1.61). Maximal running speed achieved by CD averaged 27.7 ± 1.7 km·h^−1^ (Figure 2).

### Accelerometry

**Figure 3:**
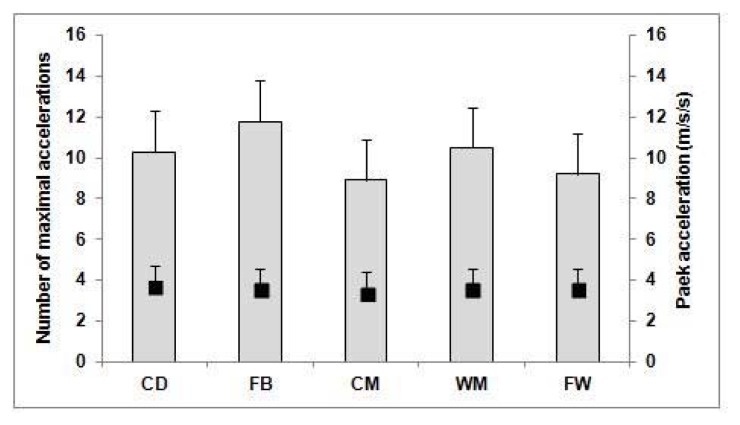
The number of maximal accelerations (>2.78 m·s^−2^) and peak acceleration values (m·s^−2^) during a match in relation to a playing position CD: Central Defenders. FB: Fullbacks. CM: Central Midfielders. WM: Wide Midfielders. FW: Forwards.

The players carried out, on average, 581 ± 59 accelerations per match (Table 2). Central defenders carried out more accelerations than FW (*p* < 0.001; effect size: 1.65), WM (*p* < 0.001; effect size: 1.48), and FB (*p* < 0.05; effect size: 1.17). The average number of maximal accelerations per match was 10.1 ± 5.2, with no significant differences between playing positions (Figure 3). The peak acceleration value during the match averaged 3.6 ± 0.4 m·s^−2^, with no significant differences between playing positions (Figure 3).

### Heart Rate

The mean heart rate during a match was 165 ± 11 beats·min^−1^, which corresponded to 84.7 ± 5.1% of the individual HR_max_. Table 3 shows the heart rate distribution into different cardiac categories. There were no significant differences in any of the categories between playing positions.

## Discussion

This study presents a physical profile of Spanish elite soccer players according to their playing position, obtained during pre-season friendly matches. Despite being a frequent study topic during the last years, the novelty of this research relies on the use of GPS technology for the determination of physical match demands. Positional differences were detected in total distance covered and by high-intensity activities, suggesting the need to develop specific training programs in accordance to match demands. When comparing results from this study with data available in the literature, important differences were detected in the amount of distance covered by sprinting, which suggests that caution should be taken when data obtained with the GPS and other motion analysis systems are interchanged, specifically regarding high-intensity activities. In addition, the determination of accelerations can help enhance knowledge on the high-demanding moments that occur during training and match-play.

During the last decade, GPS technology has become a very useful tool to monitor training workloads in team sports (Larsson, 2003). Alongside with the calculation of distances covered and speed intensities during the displacements, modern GPS units integrate accelerometer and heart rate data (Carling et al., 2008), helping to draw a more precise picture of the situation. However, due to the Laws of the Game regulations, the GPS and/or any other electronic material are not allowed to be used in competition and, hence, investigations have to be carried out on friendly matches or at lower competitive standards (Impellizzeri et al., 2005).

Distance covered during friendly matches by Spanish top-class players monitored with GPS devices averaged 10.8 km, which is practically an identical value than that calculated with a multi-camera match analysis system in official “La Liga” matches (Dellal et al., 2011). Positional differences were detected in total distance covered, as WM covered the greatest distance followed by CM, FW, FB, and CD. This descendent sequence is also characteristic from other studies carried out with top-class soccer players taking part in the Spanish, Italian and English leagues, where wide and central midfielders covered the greatest distances followed by FW and FB, whereas CD covered the least distances (Bradley et al., 2009; Dellal et al., 2011; Di Salvo et al., 2007; Rampinini et al., 2007).

Distance covered at high-intensities has been traditionally identified as a key performance indicator of physical match performance (Mohr et al., 2003) and has been related to training status (Krustrup et al., 2003). Wide midfielders ran the greatest distances at high-intensities (>14.4 km·h-1), whereas CD ran the least, which is consistent with findings from previous studies (Bradley et al., 2009; Dellal et al., 2011; Di Salvo et al., 2007). Interestingly, CM covered the greatest distance by running (14.4–19.8 km·h-1) although they sprinted the lowest distance (25 km·h-1) of all the playing positions. These data are reinforced by the fact that CM achieved the lowest peak running speed during the matches. Altogether, this should have practical consequences on training prescription as CM need to stimulate their ability to produce frequent moderate to high-intensity periods of exercise rather than isolated maximal explosive bouts. On the other hand, FB, WM, and FW covered the greatest distances by sprinting and achieved the highest running speeds. However, even though this tendency has been reported elsewhere (Bradley et al., 2009; Dellal et al., 2011; Di Salvo et al., 2007), it is important to highlight inter-studies differences in the absolute sprinting values presented. As an example, players examined in the current study covered 88% and 60% more distance by sprinting than those studied by Di Salvo et al. (2010) and Bradley et al. (2009), respectively, even though similar speed thresholds to define this effort category were employed in all the investigations. These differences cannot be solely explained by the standard of the competition as the present study examined friendly matches whereas UEFA international club and Premier League matches were observed by Di Salvo et al. (2010) and Bradley et al. (2009), respectively. This suggests that sports scientists should be cautious when comparing data obtained with different match analysis systems (Harley et al., 2011; Randers et al., 2010). Even though there is no “gold standard” validation method for time-motion analysis systems (Carling et al., 2008), it appears that differences between methodologies increase as the speed of the displacements of the players do so. For instance, while distance covered between studies can be similar, important differences arise when movements are performed at higher intensities, especially exceeding 20 km·h-1 (Coutts and Duffield, 2010).

The integration of accelerometers in GPS devices has enabled to obtain information about the number of accelerations that the players carry out during a match. The frequency of accelerations registered in the present study averaged 581, with CD performing the greatest number of them. However, important differences between positions were detected at intensities below 1.5 m·s-2. To further examine high-intensity exercise, we selected maximal accelerations for analysis, which were considered as those exceeding 2.78 m·s-2 (Aughey, 2010). The players examined in the current study presented a mean value of 10 maximal accelerations per match. To our knowledge, Varley et al. (2011) were the first ones to assess these maximal accelerations during competitive soccer, reporting a frequency of 54 per match. This parameter shows a wide range in different team sports going from 10 accelerations per match in rugby (>2.5 m·s-2; Cummins et al., 2013) to 96 in Australian football (>2.78 m·s-2; Aughey, 2010). It should be respected that the determination of accelerations might still have unresolved methodological issues, related not only to the kind of accelerometer and sampling rate used (Cummins et al., 2013), but also to the way data are mathematically treated. In this sense, Bucheit et al. (2013) recently showed that this treatment (software updates) could have a significant effect on the calculation of accelerations. The capacity to accelerate and decelerate plays a critical role in elite soccer as they represent high energy demanding activities. This has led to the necessity of redefining the concept of high-intensity exercise on the basis of the actual metabolic power rather than on speed itself (Osgnach et al., 2010). For instance, significant differences have been found between distance covered at high-intensities and estimated equivalent metabolic power during elite soccer training activities (Gaudino et al., 2013).

Heart rate measurement has been used to estimate energy expenditure in friendly matches, as heart rate monitors are not allowed to be worn during competitive games (Drust et al., 2007). The mean heart rate during the game corresponded to 85% of the individual HRmax, which is in line with previous studies (Dellal et al., 2012). Heart rate data can be used to provide an overall indicator of the exercise intensity and predict intense match periods (Mallo et al., 2009), although it requires from other physical parameters to obtain a global indicator of the competition intensity.

The findings presented here are limited by some methodological issues. First, data were obtained from 11-a-side friendly matches and not from official competition. Due to the impossibility of using the GPS during official matches, this limitation was impossible to overcome during the experimental design of the investigation. The second limitation comes from the sampling frequency of the GPS (1 Hz). An increment in this rate would probably increase the accuracy of the measurements, especially regarding high-speed efforts (Cummins et al., 2013). The development of new GPS models will facilitate the publication of data with the GPS operating at higher sampling rates.

The results of this study show that physical demands are influenced by the playing position of the players and should be used to prescribe specific training plans in accordance to them. Wide midfielders experienced the greatest physical requirements during the game, both in terms of total distance covered and by very high-intensity running. Sprinting was predominant in FW and FB, who showed moderate values of total distance covered during the games. The physical performance of CM was characterized by covering a high overall distance, especially at moderate to high speeds (jogging and running). Finally, CM covered the lowest total distance and by high-intensities. An additional finding from this study was the necessity to be cautious when comparing physical data obtained with different motion analysis systems as important differences were detected between our results and previous data calculated with semi automatic tracking systems in players of a similar standard of competition.

## Figures and Tables

**Table 1:** Distance covered (m) at different speeds during a match in relation to a playing position

	Standing Still	Walking	Jogging	Running	High-Speed Running	Sprint	Total
CD	101±192	4323±409	3709±501	1483±410	343±96***	247±152**	10206±1067
FB	97±124	4456±374	3535±573	1433±363#	437±153	494±249	10452±1063
CM	140±276	4077±414	4256±621$	2079±452##	396±135&	208±132###	11154±1117
WM	122±334	4290±339	4015±839	1878±583	533±182	482±183	11321±1238^*^
FW	69±53	4370±247	3605±649	1715±352	461±114	505±188	10726±879
Average	107±226	4299±377	3839±697	1726±502	437±154	385±223	10793±1153

Significant difference (p < 0.05) between WM and CD;

# Significant difference (p < 0.05) between FB and WM;

$ Significant difference (p < 0.05) between CM and FB and FW;

& Significant difference (p < 0.05) between CM and WM;

** Significant difference (p < 0.01) between CD and FB, CM, WM and FW;

## Significant difference (p < 0.01) between CM and FB;

*** Significant difference (p < 0.001) between CD and WM;

### Significant difference (p < 0.001) between CM and FB, WM and FW.

CD: Central Defenders. FB: Fullbacks.

CM: Central Midfielders. WM: Wide Midfielders. FW: Forwards.

**Table 2:** The number of accelerations at different intensities during a match in relation to a playing position

	<1.0 m·s^−2^	1.0–1.5 m·s^−2^	1.5–2.0 m·s^−2^	2.0–2.5 m·s^−2^	>2.5 m·s^−2^	Total
CD	385±50*	123±21	70±16	33±8	18±6	629±46$$$
FB	358±43	106±12	63±22	30±10	19±9	575±47$
CM	337±55	127±25	73±20	35±7	17±7	590±70
WM	327±48	112±23	62±10	35±8	21±6	557±49
FW	334±35	100±29#	68±19	30±9	19±8	553±47
Average	348±51	114±24	168±73	33±8	19±7	581±59

* Significant difference (p < 0.05) between CD and CM, WM, and FW.

# Significant difference (p < 0.05) between FW and CD, and CM.

$ Significant difference (p < 0.05) between FB and CD.

$$$ Significant difference (p <0.001) between CD and WM, and FW.

CD: Central Defenders. FB: Fullbacks. CM: Central Midfielders.

WM: Wide Midfielders. FW: Forwards.

**Table 3:** The mean heart rate (%HR_max_) and percentage of time spent at different intensities (%HR_max_) during a match in relation to a playing position

	Average (%HR_max_)	<60% HR_max_	61–70% HR_max_	71–80% HR_max_	81–90% HR_max_	91–95% HR_max_	>95% HR_max_
CD	85.1 ± 5.0	0.7 ± 1.9	5.5 ± 7.0	11.0 ± 8.1	40.7 ± 9.5	39.0 ±13.9	3.2 ± 3.5
FB	83.0 ± 5.2	0.2 ± 0.4	9.2 ± 7.5	14.3 ± 8.6	45.2 ± 13.2	28.6 ± 14.9	2.6 ± 3.6
CM	86.0 ± 4.5	0.4 ± 1.2	3.8 ± 3.1	14.1 ± 8.9	41.1 ± 10.0	37.7 ± 11.1	2.9 ± 3.8
WM	85.3 ± 5.1	1.5 ± 2.8	7.7 ± 8.0	13.7 ± 10.0	41.4 ± 11.2	30.1 ±16.4	4.9 ± 4.2
FW	84.3 ± 5.6	1.0 ± 2.7	7.0 ± 6.4	12.5 ± 6.9	44.1 ± 12.2	31.1 ± 12.2	4.0 ± 5.5
Average	84.7 ± 5.1	0.7 ± 2.1	6.6 ± 6.8	13.0 ± 8.4	42.5 ± 11.2	33.6 ± 14.2	3.5 ± 4.2

CD: Central Defenders. FB: Fullbacks. CM: Central Midfielders. W

M: Wide Midfielders. FW: Forwards
